# Combined urine metabolomics and 16S rDNA sequencing analyses reveals physiological mechanism underlying decline in natural mating behavior of captive giant pandas

**DOI:** 10.3389/fmicb.2022.906737

**Published:** 2022-09-02

**Authors:** Ming-yue Zhang, Xue-ying Wang, James Ayala, Yu-liang Liu, Jun-hui An, Dong-hui Wang, Zhi-gang Cai, Rong Hou, Kai-lai Cai

**Affiliations:** ^1^Chengdu Research Base of Giant Panda Breeding, Chengdu, China; ^2^Sichuan Key Laboratory of Conservation Biology for Endangered Wildlife, Chengdu, China; ^3^Sichuan Academy of Giant Panda, Chengdu, China

**Keywords:** captive giant panda, mate choice, metabolomics, 16s rDNA sequencing, decline in mating behavior

## Abstract

The decline in natural mating behavior is the primary reason underlying in the poor population growth of captive giant pandas. However, the influencing factors and underlying mechanisms remain unclear to data. It is speculated that the decline in natural mating behavior could be related to the psychological stress caused by captivity, which restricts their free choice of mates. In order to test this hypothesis, we performed urinary metabolomics analysis using Ultra-High-Performance Liquid Chromatography-Mass Spectrometry (UHPLC/-MS) combined with 16S rDNA sequencing for exploring the physiological mechanism underlying the decline in the natural mating behavior of captive giant panda. The results demonstrated that the decline in mating ability could be related to abnormalities in arginine biosynthesis and neurotransmitter synthesis. Additionally, the relative abundance of bacteria from the Firmicutes, Proteobacteria, and Actinobacteria phyla and the *Acinetobacter, Weissella*, and *Pseudomonas* genus was significantly reduced in the group with low natural mating behavior. These findings imply that the inhibition of arginine synthesis induced by environmental changes could be related to the poor libido and failure of mate selection in captive giant pandas during the breeding period. The results also demonstrate the relationship between the altered urinary microbes and metabolites related to arginine and neurotransmitter synthesis. These findings may aid in understanding the mechanism underlying environment-induced mate selection in captive giant pandas and propose a novel strategy for determining the sexual desire of giant pandas based on urinary microbes. The method would be of great significance in improving the natural reproductive success rate of captive giant pandas.

## Introduction

The decline in natural mating behavior is a serious issue faced by the population of captive giant pandas (*Ailuropoda melanoleuca*) (Xie, 2016), and mate choice is an important factor in the natural mating process of giant pandas (Lumley et al., 2015; Martin-Wintle et al., 2015, 2018). Captive giant pandas often demonstrate low natural reproductive efficiency; however, the mechanism underlying the decline in instinctive mating behavior remains unclear. The giant panda is a solitary animal, distributed in the mountains of Sichuan, Shaanxi, and Gansu provinces in China (Schaller et al., 1985). Years of behavioral observations have demonstrated that both wild and captive pandas show strong mate selection behavior (Martin-Wintle et al., 2015; Owen et al., 2016). Contrary to wild giant pandas that have a free choice of mates, captive giant pandas are often paired one-on-one. The present captive breeding model severely limits the free mating choice of giant pandas in captivity, resulting in the lack of strong sexual desire for the opposite sex in the majority of captive giant pandas during the breeding season, which eventually leads to the failure of natural mating (Martin-Wintle et al., 2020; Zhang et al., 2021b). Although large spaces and environmental enrichment can effectively improve the expression of estrus behavior in captive giant pandas (Peng et al., 2007), the low natural mating efficiency of captive giant pandas remains to be solved (Martin-Wintle et al., 2018; Zhang et al., 2021b). Our years of experience have revealed that in 90% of cases, the low natural reproductive success rates of captive giant pandas is attributed to the failure of mate selection resulting from the inability to freely choose a mate. The failure of mate choice is not due to the absence of estrus behavior or the size of the population. Therefore, investigating the mechanism underlying the decline in the natural reproductive behavior from the perspective of mate selection based on environmental adaptation can provide a novel avenue of research. Giant pandas endure the pressure of environmental changes during *ex situ* conservation (Wei et al., 2015; Ciminelli et al., 2021; Zhang et al., 2021a), posing great challenges to their environmental adaptability (The captive environment is capable of meeting the various physiological needs of giant pandas) (Martin-Wintle et al., 2020; Zhang et al., 2021b). Previous studies have suggested that captive giant pandas may suffer from long-term stress and even psychological discomfort in captivity. Moreover, environmental discomfort suppresses the expression of reproductive behavior and impairs the reproductive performance of giant pandas (Martin-Wintle et al., 2020). We therefore speculate that the psychological frustration caused by long-term environmental constraints and failure of mate selection appears to further aggravate the inability of mate selection (Zhang et al., 2004, 2022); however, direct evidence is lacking in this regard. This study aimed to employ a multi-omics strategy for elucidating the mechanism underlying the decline in the natural reproductive behaviors of captive giant pandas from the perspective of environmental adaptability.

Metabolomics is a branch of science concerned with the types, quantities, and alterations of endogenous metabolites induced by external stimuli, pathophysiological changes, and gene mutations (Cao et al., 2020). Metabolomics studies typically analyze the differences in the levels of different metabolites between experimental and control groups for identifying the altered metabolites, which aids in the biomarker screening. Metabolomics analyses also aid in studying the biological processes associated with the altered metabolites (by reverse investigation of regulating enzymes and genes through metabolic pathways), and elucidate the underlying biological mechanism (Nicholson and Lindon, 2008). The majority of potential biomarkers reported so far are derived from urine samples due to the fact that urine can be conveniently analyzed and can be collected non-invasively. Therefore, urine samples are frequently used for identifying the altered metabolites in various common and rare diseases (Collino et al., 2013; Khamis et al., 2017). Since its discovery and confirmation, the human urinary microbiome has been found to be closely related to the development of several diseases (Bajic et al., 2018; Fok et al., 2018; Popovic et al., 2018), even infertility in humans (Lundy et al., 2021). For instance, specific bacterial communities, including Lactobacillus sp., have been found in healthy urinary tracts. Alterations in the urinary microbiome, such as a higher abundance of *L*. sp., *Klebsiella* sp., *Corynebacterium* sp., and *Staphylococcus* sp., have been observed in certain urologic disorders, including urinary incontinence, urologic cancers, interstitial cystitis, neurogenic bladder dysfunction, sexually transmitted infections, and chronic prostatitis/chronic pelvic pain syndrome (Aragón et al., 2018). The 16S rDNA refers to the DNA sequence that encodes the ribosomal 16S rRNA molecule in the bacterial genome, that is, the gene encoding the bacterial 16S rRNA is referred to as the 16S rDNA. The 16S rDNA sequence comprises 10 conserved regions and 9 hypervariable regions, of which the conserved regions have little difference among bacteria, while the hypervariable regions have genus- or species-specificity and exhibit unique kin-specific differences (Wolfe and Brubaker, 2019). Therefore, the 16S rDNA sequence can be used as a characteristic nucleic acid sequence for identifying bacterial species and is considered to be the most suitable indicator in bacterial phylogenetic analyses and taxonomic identification. The 16S rDNA amplicon sequencing technology has become an important technique for studying the composition and structure of microbial communities in environmental samples (Watanabe and Koga, 2009; Youssef et al., 2009; Caporaso et al., 2011; Hess et al., 2011).

Based on the aforementioned reasons, and combined with the key role of urinary markers in the mate choice of captive giant pandas, we speculate that certain urinary biomarkers may act as important influencing substances that led to the failure of mate choice in captive giant pandas, resulting in the decline in their natural mating behavior. Therefore, this study aimed to use non-target Ultra-High-Performance Liquid Chromatography-Mass Spectrometry (UHPLC-MS) for metabolomics analysis and a 16S rDNA sequencing technology for studying the differences in the levels of urinary metabolites of captive giant pandas during peak estrus. The study also aimed to identify the alterations in urinary microbial species and abundance, and explore the correlation between the altered urinary microbiota and levels of altered metabolites. In order to identify the key bacterial species and metabolites, enrichment analysis of the metabolic pathways and functional annotation of bacterial flora was performed, which revealed the physiological mechanism underlying the decline in the success rate of mate choice and natural mating behavior of captive giant pandas.

## Materials and methods

### Animals and ethics statement

In this study, 12 captive giant pandas from the Chengdu Research Base of Giant Panda Breeding (Panda Base) were selected as research subjects. The animals are divided into two groups: NM group, six had successful natural mating experience (able to produce offspring through natural mating after adulthood), and AI group, another six adult giant pandas did not have successful natural mating experience (did not produce offspring through natural mating after adulthood). The study protocol was approved by the Institutional Animal Care and Use Committee of Chengdu Research Base of Giant Panda Breeding (approved number: 2020013).

### Urine sampling

Previous research studies at the Panda Base have demonstrated that the appearance of the urinary luteinizing hormone peak indicated the timing of ovulation and can be used to determine the appropriate time for natural mating (Cai et al., 2017). After years of positive behavioral training by the foreign animal behavior expert, James Ayala, and breeders at Panda Base, we have successfully trained giant pandas to urinate at a fixed location, which ensured that the urine samples used in this experiment were devoid of contaminants from the external environment at the time of sampling. In this study, urine samples from the 12 captive adult giant pandas were collected during peak estrus in the breeding season (February to April) in 2021. The specific groupings are depicted in Table 1. The urine samples were collected with a syringe from the clean floor of an enclosure, once every morning and afternoon during estrus, and every 2 h during the peak period, and transported to the laboratory on ice. The samples were stored at −80°C until further analyses. The samples were subjected to 16S rDNA sequencing and metabolomics analysis with UHPLC-MS, according to the manufacturer's instructions (Shanghai Applied Protein Technology).

**Table 1:** Experimental grouping.

**Group**	**Name**	**Studbook**	**Birth**	**Sex**	**Wild or Captive**	**Whether can produce cubs naturally**
NM	Gong Zi	711	2008	Male	Captive	Yes
NM	ABao (Lou)	703	2007	Male	Wild	Yes
AI	Ying Ying	724	2008	Male	Captive	No
AI	Xi Lan	731	2008	Male	Captive	No
NM	Mei Lan	649	2006	Male	Captive	Yes
AI	Xing Bang	614	2005	Male	Captive	No
NM	Zhao Mei	990	2010	Female	Wild	Yes
AI	Ni Da	995	2015	Female	Captive	No
AI	Ya Zai	637	2006	Female	Captive	No
NM	ABao (USA)	801	2010	Female	Captive	Yes
NM	Ya Yun	796	2010	Female	Captive	Yes
AI	Mei Huan	871	2013	Female	Captive	No

The animals are divided into two groups: NM group, six had successful natural mating experience (able to produce offspring through natural mating after adulthood), AI group, and another six adult giant pandas did not have successful natural mating experience (did not produce offspring through natural mating after adulthood).

### Liquid chromatography-mass spectrometry/mass spectrometry (LC-MS/MS) analysis

#### Sample preparation

Urine samples were collected in 5-mL vacutainer tubes containing ethylene diamine tetra acetic acid (EDTA) as the chelating agent, following which the samples were centrifuged at 1,500 × g for 15 min (at 4°C). Then, 150 μl aliquots of urine samples were stored at −80°C until UHPLC-quadrupole time-of-flight (Q-TOF)/MS analysis. The samples were slowly thawed at 4°C, following which an appropriate quantity of the samples was added to a pre-cooled solution of methanol/acetonitrile/water (2:2:1, v/v), mixed by vortex agitation, sonicated at a low temperature for 30 min. The samples were then centrifuged at 14,000 × g for 20 min at 4°C, following which the supernatant was removed and dried in a vacuum. Then, 100 μL of an aqueous solution of acetonitrile (acetonitrile:water = 1:1, v/v) was added for reconstituting the sample, which was re-mixed by vortex agitation and centrifuged at 14,000 × g for 15 min at 4°C. The sample was finally removed for serum injection analysis.

#### Chromatography-MS

The samples were analyzed using an UHPLC system (1290 Infinity LC, Agilent Technologies) coupled to a Q-TOF platform (AB Sciex TripleTOF 6600; Shanghai Applied Protein Technology Co., Ltd.). The chromatographic conditions were as follows: the samples were separated by an Agilent 1290 Infinity LC UHPLC HILIC column; column temperature 25°C; flow rate: 0.5 mL/min; injection volume: 2 μL; composition of mobile phase A: water + 25 mM ammonium acetate + 25 mM ammonia water; mobile phase B: acetonitrile. The gradient elution program is as follows: 0–0.5 min, 95% phase B; 0.5–7 min, phase B was linearly changed from 95 to 65%; 7–8 min, phase B was linearly changed from 65 to 40%; 8–9 min, phase B was maintained at 40%; 9–9.1 min, phase B was linearly changed from 40 to 95%; and 9.1–12 min, phase B was maintained at 95%. The samples were placed in an autosampler at 4°C during the analysis. The influence due to fluctuations in the instrumental detection signal was excluded by the continuous analysis of the samples in a random manner. Quality control (QC) samples were inserted into the sample queue for monitoring and evaluating the stability of the system, and ensuring the reliability of the experimental data. The conditions of Q-TOF/MS were as follows: the primary and secondary spectra of the samples were collected using an AB Triple TOF 6600 mass spectrometer. The samples were separated on an Agilent 1290 Infinity LC UHPLC system, following which MS was performed on a Triple TOF 6600 mass spectrometer (AB SCIEX) using electrospray ionization (ESI) in positive and negative ion modes. The settings of the ESI source parameters were as follows: nebulizer gas auxiliary heater gas 1 (Gas1), 60; auxiliary heater gas 2 (Gas2): 60; curtain gas (CUR): 30 psi; ion source temperature: 600°C; spray voltage (ISVF) ± 5,500 V (both positive and negative modes); primary mass-to-charge ratio detection range: 60–1,000 Da; secondary product ion mass-to-charge ratio detection range: 25–1,000 Da, primary MS scan accumulation time: 0.20 s/spectra; and secondary MS scan accumulation time 0.05 s/spectra and mass spectra were acquired in data-dependent acquisition mode (IDA) using the peak intensity value screening mode; declustering voltage (DP): ±60 V (both positive and negative modes); collision energy: 35 ± 15 eV. The settings in IDA were as follows: dynamically excluded isotope ion range: 4 Da, 10 fragment spectra were collected per scan (Blaženović et al., 2018).

#### Data processing

The raw data obtained in Wiff format (wiff.scan files) were converted to MzXML format using ProteoWizard. The XCMS software was then used for aligning the peaks, correcting the retention time, and extraction of peak area. The data extracted by XCMS were first used to identify the structures of the metabolites, following which the data were preprocessed. Data analysis was finally performed after evaluating the quality of the experimental data (Wen et al., 2017).

#### Statistical analyses

Data analyses were performed by univariate statistical analysis, multi-dimensional statistical analysis, differential metabolite screening, differential metabolite correlation analysis, Kyoto Encyclopedia of Genes and Genomes (KEGG) pathway analysis, and other analyses. The statistical analyses are described in detail in our previous reports (Zhang et al., 2022).

### 16S rDNA amplicon sequence analyses

#### DNA extraction and amplification with polymerase chain reaction (PCR)

In order to avoid environmental contamination, the genomic DNA from the urine samples and the reagent-only control sample was extracted on a sterile operating table. The total genomic DNA was extracted from the samples using the CTAB/SDS method (Ma et al., 2020). The concentration and purity of the DNA was determined using 1% agarose gels. Selected V3-V4 variable regions were amplified with PCR using specific primers with barcodes and high-fidelity DNA polymerase based on the selected sequenced regions. The PCR products were detected by gel electrophoresis using 2% agarose gels, and the target fragments were cut and recovered using an AxyPrepDNA gel recovery kit. Based on the preliminary quantitative results of electrophoresis, the products recovered from PCR amplification were detected and quantified with a QuantiFluor™-ST blue fluorescence quantitative system (Promega Company), and the corresponding proportions were mixed according to the amount of sample sequenced. Library was constructed using an NEB Next® Ultra™ DNA Library Prep kit. Library was rechecked using an Agilent Bioanalyzer 2100 and Qubit, and the library was sequenced after quality check.

#### Data analyses

Paired-end reads from the original DNA fragments were merged using FLASH, a very rapid and accurate analysis tool that is designed to merge paired-end reads when at least some portion of the reads overlap with the read generated from the opposite end of the same DNA fragment. Paired-end reads were assigned to each sample according to the unique barcodes. Sequence analyses were performed using the UPARSE software package, with the UPARSE-OTU and UPARSE-OTUref algorithms. In-house Perl scripts were used for analyzing the alpha (within sample) and beta (among samples) diversity. Sequences with ≥97% similarity were assigned to the same operational taxonomic units (OTUs). We selected a representative sequence for each OTU and used the Ribosomal Database Project (RDP) classifier for annotating the taxonomic information of each representative sequence. The alpha diversity was determined by rarifying the OTU table and calculation of three metrics, namely, (1) Chao1, which provides a measure of estimated species abundance; (2) observed species, which estimates the number of unique OTUs in each sample; and (3) the Shannon index. Rarefaction curves were generated based on these three metrics. The relative abundance of bacterial diversity at phylum to species levels was graphically represented and visualized using a Krona chart. Cluster analysis was preceded by principal component analysis (PCA) for reducing the dimensionality of the original variables using the QIIME software package. QIIME calculates both the weighted and unweighted UniFrac distances, which are phylogenetic measures of beta diversity. The unweighted UniFrac distance was used for principal coordinate analysis (PCoA) using the unweighted pair-group method with arithmetic mean (UPGMA) for clustering. The principal coordinates were obtained from PCoA, which aided in visualizing the coordinates of complex, multidimensional data. PCoA also transformed the distance matrix to a new set of orthogonal axes, in which the first principal coordinate represents the factor with the maximum variation, and the second principal coordinate represents the factor with the second maximum variation, and so on. UPGMA clustering is a hierarchical clustering method using average linkage and can be used to interpret distance matrices.

#### Statistical analysis

The differences between the individual taxonomic abundances of the two groups were determined using the STAMP software. The LEfSe method was used for quantitative analysis of biomarkers within different groups. This method is designed to analyze data in which the number of species is much higher than the number of samples, and is used to provide biological class explanations for establishing statistical significance, biological consistency, and effect-size estimation of predicted biomarkers. Adonis analysis was performed based on Bray-Curtis dissimilarity distance matrices for identifying the differences between the microbial communities of the two groups.

### Correlation analysis

The relative abundances of the bacterial groups in the 12 urine samples, showing significant differences at the genus level (LEfSe linear discriminant analysis (LDA)>2 and *p*-value < 0.05), as revealed by 16S rDNA amplicon sequence analysis of the 12 experimental samples, were compared with the results of metabolomics analysis. The expression levels of 38 significantly different metabolites (variable importance in the projection (VIP) >1 and *t*-test *p*-value < 0.05) were organized in a table as input files for subsequent analyses. The correlation coefficient between the significantly altered bacterial groups and the metabolites showing significantly altered urinary levels in the experimental samples was determined using Spearman's correlation method. A heatmap matrix was constructed and hierarchical clustering, correlation network analysis, and other statistical analyses were performed using R scripts and Cytoscape software, for analyzing the interactions between the urinary microbiota and metabolites from multiple perspectives (Cribbs et al., 2016).

## Results

### Bioinformatics analysis of differential metabolites

The quality of the data was first evaluated prior to data analysis. The system stability of the project was analyzed and evaluated by comparing the QC and sample spectra, and PCA analysis. The total ion chromatogram (TIC) of the QC samples was compared for spectral overlap. The experimental results demonstrated that the response intensity and retention time of each chromatographic peak basically overlapped, indicating that the variation caused by instrumental error was small during the experimental process. The extracted peaks extracted from the experimental and QC samples were also subjected to PCA analysis. The experimental results demonstrated that the QC samples in the positive and negative ion modes were closely clustered together, indicating that the experiment has good repeatability. The metabolites that were significantly different between the two samples were identified by univariate data visualization (Supplementary Figures S1A,B). We subsequently used multivariate statistical methods, including principal component analysis (PCA), partial least-squares discriminant analysis (PLS-DA), and orthogonal partial least-squares discriminant analysis (OPLS-DA), for performing dimensionality reduction analysis of the collected multi-dimensional data by preserving the original information to the maximum extent. In this study, the data was cross-validated seven-fold using the multivariate statistical model constructed with PCA, PLS-DA, and OPLS-DA. The main parameter in the PCA model is *R*^2^*X*; a value closer to 1 indicates that the model is more stable and reliable. The *R*^2^*X* values of the NM-AI group in positive and negative ion modes were 0.557 and 0.603, respectively. The evaluation parameters of the PLS-DA model for the NM-AI group were calculated (positive ion mode: *R*^2^*Y* = 0.996, *Q*^2^ = 0.977; negative ion mode: *R*^2^*Y* = 0.997, *Q*^2^ = 0.840). The OPLS-DA model was also constructed in this study. The values of the parameters used for evaluating the model for the NM-AI group were also determined (positive ion mode: *R*^2^*Y* = 0.996, *Q*^2^ = 0.950; negative ion mode: *R*^2^*Y* = 0.997, *Q*^2^ = 0.954). The results of multivariate statistical analyses also revealed that the spectrum of metabolites in the AI-group underwent significant alterations or were even partially disordered compared with that of the NM-group.

In order to detect the changes in the concentration of the metabolites between the NM and AI groups, strict metabolite screening criteria, namely, OPLS-DA VIP>1 and *p*-value < 0.05, were used for identifying a total of 136 metabolites with altered urinary levels. Of these, 64 metabolites were identified in the positive mode and 72 metabolites were identified in the negative mode. The major metabolites with altered urinary levels were categorized as organoheterocyclic compounds (kynurenic acid, adenine, and others), organic acids and derivatives (DL-glutamic acid, glutamine, aspartic acid, sulfoacetic acid, and other compounds), lipids and lipid-like molecules, benzenoids, phenylpropanoids, and polyketides (Supplementary Figures S1C,D). Of these, the urinary levels of 43 kinds of urinary metabolites, including DL-glutamic acid and glutamine, increased significantly in the AI group (*p* < 0.05), while the levels of 93 kinds of urinary metabolites, including aspartic acid, kynurenic acid, and sulfoacetic acid significantly decreased in the AI group, compared with those of the NM group (*p* < 0.05) (Supplementary Figures S1C,D).

**Figure 1:**
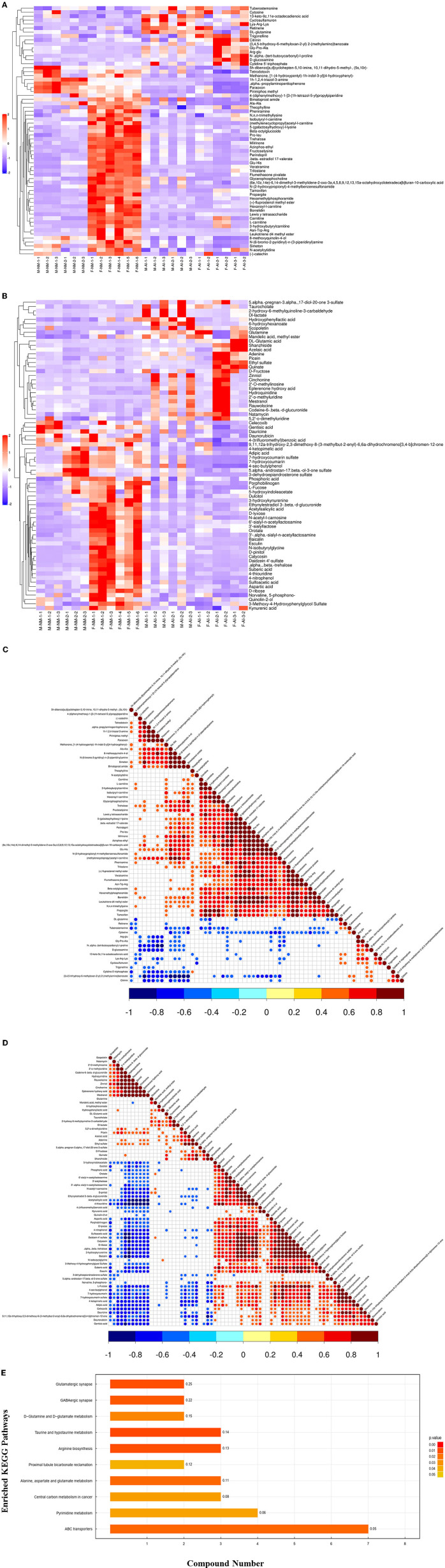
Bioinformatics analysis of differential metabolites. **(A)** Significant difference metabolite hierarchical clustering results in positive ion mode. **(B)** Significant difference metabolite hierarchical clustering results in negative ion mode. **(C)** Relevance among significant difference metabolites. **(D)** Relevance among significant difference metabolites. **(E)** KEGG pathway enrichment analysis of differentially expressed metabolites.

In order to determine the relationship between the samples and identify the differences in the expression patterns of the metabolites in different samples more comprehensively and intuitively, the expression levels of the metabolites in all the samples and differential metabolites were subtracted from the average value of the corresponding groups, and subsequently divided by the root mean square of the group for normalization. The distance matrix was subsequently calculated, and the hierarchical clustering method was used for cluster analysis (Figures 1A,B). We observed relevance among the metabolites with significantly altered urinary levels (Figures 1C,D). The urinary metabolites were subjected to KEGG metabolic pathway enrichment analysis. KEGG pathway enrichment analysis of the differentially expressed metabolites by Fisher's exact test revealed significant changes in important pathways, including the pathways of taurine and hypotaurine metabolism, arginine biosynthesis, glutamatergic synapse, GABAergic synapse, ABC transporters, metabolism of alanine, aspartate, and glutamate, D-glutamine and D-glutamate metabolism, pyrimidine metabolism, and bicarbonate reclamation in proximal tubules (Figure 1E).

### 16S rDNA amplicon sequence analyses

#### OTU analysis and species annotation

**Figure 2:**
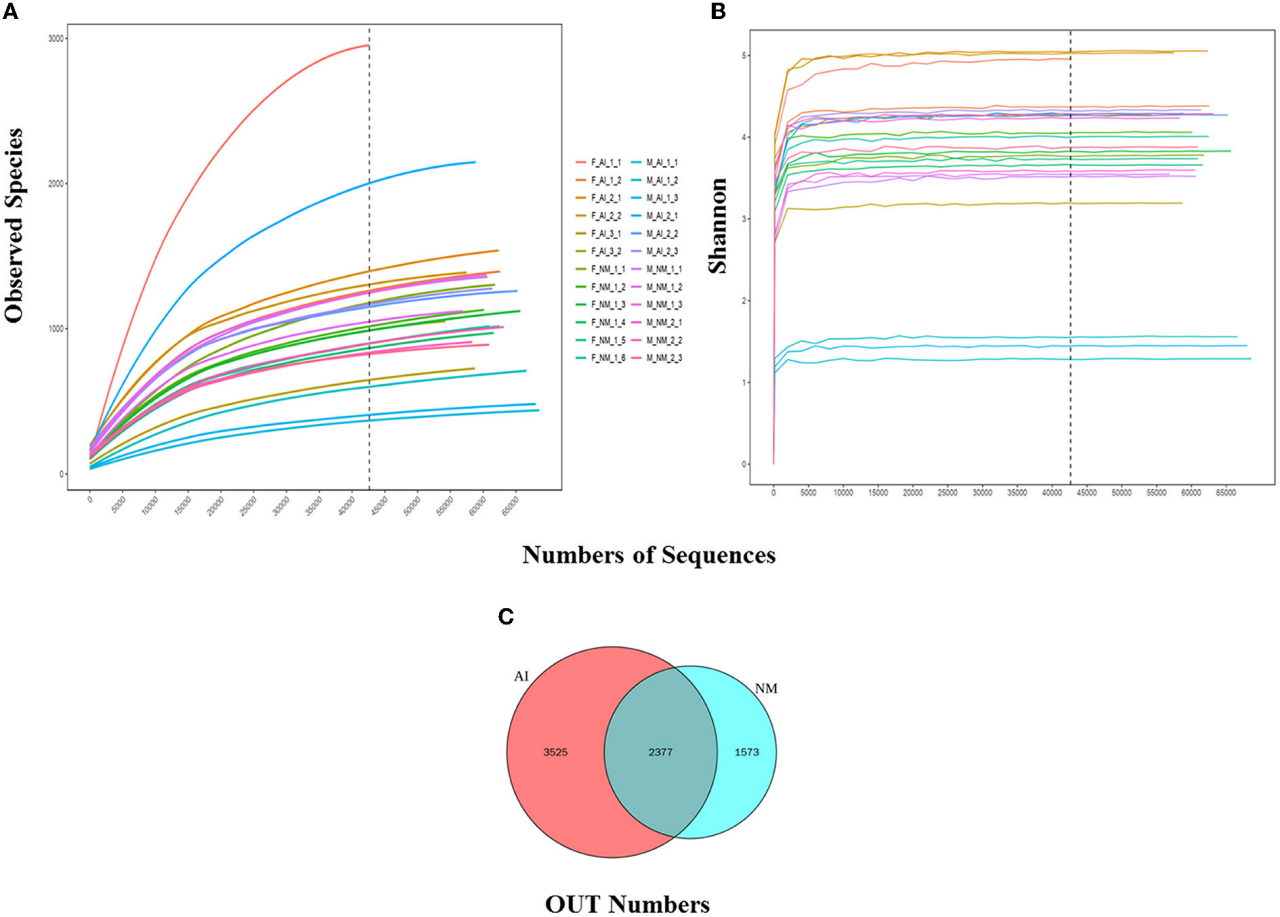
Alpha diversity. **(A)** Rarefaction Curve for different samples and different groups; Rarefaction Curve is to randomly extract a certain amount of sequencing data from a sample, count the number of species they represent, and construct a curve based on the amount of sequencing data extracted and the number of corresponding species. The dilution curve can directly reflect the rationality of the amount of sequencing data, and indirectly reflect the abundance of species in the sample. When the curve tends to be flat, it means that the amount of sequencing data is reasonable. More data will only generate a small amount of new OTUs. Otherwise, it means that continuing sequencing may generate more new OTUs. **(B)** Shannon curve is constructed according to the microbial diversity index of each sample's sequencing amount at different sequencing depths. When the curve tends to be flat, it indicates that the amount of sequencing data is large enough to reflect the vast majority of microbial information in the sample. **(C)** Venn graph of OTUs clustering. Venn graph shows the common and unique OTUs between the different groups.

The statistical data was used to process the number of sample sequences at each stage for evaluating data quality. Splicing, QC, and de-chimerism of the offline data (Raw PE) was obtained by sequencing. The values of Q20 and Q30 were above 90%, indicating that the QC data were good and met the requirements of the experimental analysis. The Rarefaction and Shannon curves revealed (Figures 2A,B) that the amount of sequencing data was reasonable and could reflect the majority of microbial information in the samples. The NM and AI groups had 2,377 identical OTUs, while 1,573 and 3,525 OTUs were unique to the NM and AI groups, respectively (Figure 2C).

#### Alpha and beta diversity

**Figure 3:**
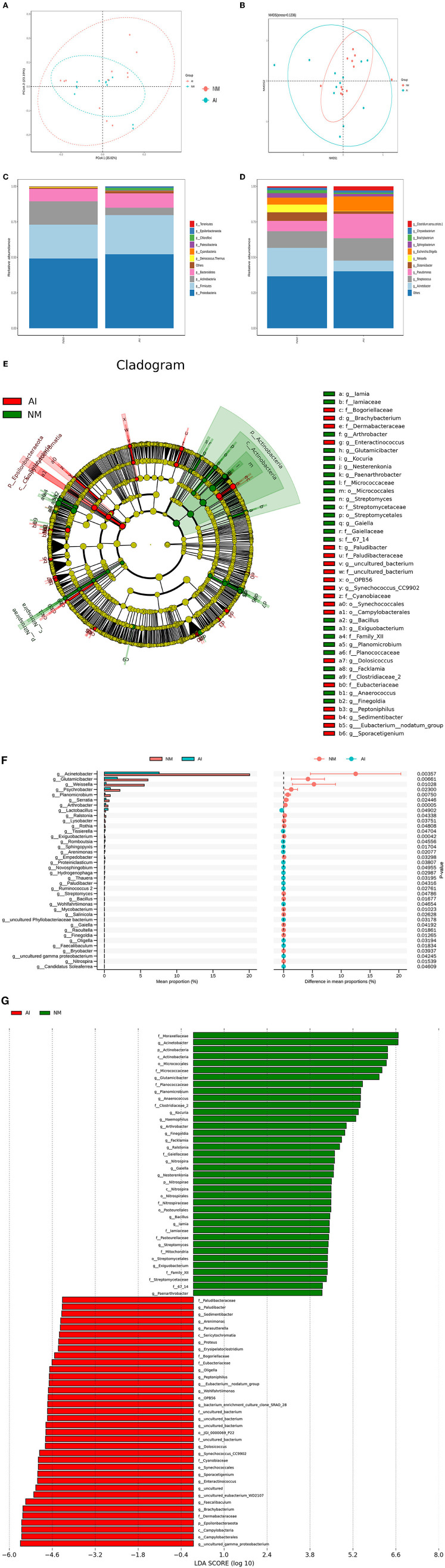
Urine microbial abundance and beta diversity in giant pandas between NM and AI group. **(A)** PCoA analysis. The abscissa represents the first principal component, the ordinate represents the second principal component, and the percentage represents the contribution to the sample difference. **(B)** Non-Metric Multi-Dimensional Scaling analysis; Each point represents a sample, the distance between points represents the degree of difference, and samples in the same group are represented by the same color. **(C)** Histogram of relative abundance of species at the level of each sample phylum. **(D)** Histogram of relative abundance of species at the level of each sample genus. Species with different metabolisms in different colors correspond to the legend on the right; the horizontal axis represents different samples or groups, and the vertical axis represents the relative abundance of different species. **(E)** Cladogram obtained by LESEF analysis. The circles radiating from the inside to the outside in the cladogram represent the taxonomic levels from phylum to genus (or species). Each small circle at a different taxonomic level represents taxonomy at that level, and the diameter of the small circle is proportional to the relative abundance. The red area and the green area represent different groups. The red nodes in the branches represent the microbial groups that play an important role in the red groups, the green nodes represent the microbial groups that play an important role in the green groups, and the yellow nodes represent the microbial groups that play an important role in the two groups. There were no microbial groups that played an important role in the group. The species names represented by the English letters in the figure are shown in the legend on the right. **(F)** LDA Score obtained by LEfSe analysis. The red and green areas in the LDA value distribution histogram represent different groups, the red nodes in the branches represent the microbial groups that play an important role in the red groups, and the green nodes represent the microbial groups that play an important role in the green groups. Only the species who's LDA Score is greater than the set value (the default setting is 2) are shown in the figure, and the length of the histogram represents the size of the LDA value. **(G)** STAMP analysis of species differences between NM group and AI group. The left figure shows the abundance ratio of different species in two samples or two groups of samples, the middle shows the difference ratio within the 95% confidence interval, the rightmost value is the *p*-value, *p*-value <0.05, indicating the difference significant.

The Chao1, Simpson, and Shannon indices were used for estimating microbial richness and diversity for characterization of microbial alpha diversity. There was no difference in the Chao1, Shannon, and Simpson indices between the NM and AI groups, indicating that there was no difference between the diversity of the microbial communities of the groups (Table 2). The weighted UniFrac index and non-metric multi-dimensional scaling (NMDS) analyses were used for measuring the beta diversity of the samples. The PCoA diagram revealed that, with the exception of the individual samples, the microbiota of the NM and AI groups were present in different regions (Figure 3A). The results of NMDS analysis revealed that the samples had significant differences between and within groups (*p* < 0.05) (Figure 3B). The differences between the community structure of the groups were analyzed by the Adonis analysis significance test (permutational MANOVA analysis). The results of Adonis analysis were: *R*^2^ = 0.0938, *p* < 0.05.

**Table 2:** Comparison of α diversity parameters between the NM group and AI group.

**Group**	**Chao**	**Shannon**	**Simpson**
NM	1270.92 ± 51.05	5.55 ± 0.11	0.92 ± 0.02
AI	1440.34 ± 197.32	5.30 ± 0.60	0.86 ± 0.04

The values are presented as the mean ± standard deviation.

#### Species composition analysis

For species annotation, the species of special interest (top 10 genera with the highest relative abundance and selected by default) were selected from the results of each sample or group for species classification tree statistics (Zhou et al., 2018) (refer Supplementary Figure S2). Based on the results of species annotation, species-level clustering was performed at each classification level (phylum, class, order, family, and genus), and the clustered data were represented as a heatmap, which is convenient for the intuitive identification of the species present in corresponding samples or groups. The results for phylum level classification are provided in Supplementary Figures S3A,B.

#### Microbial flora structure and differential analysis

Statistical analysis was performed for analyzing the composition of microbial community of the NM and AI groups at each classification level (phylum, class, order, family, genus, and species), and emphasis was placed on bacterial species with comparatively high relative abundance. We identified 10 strains at the phylum level, of which the phyla Firmicutes, Bacteroidetes, Actinobacteria, and Proteobacteria phyla were abundant in the two groups. The sum of degrees exceeded 95%. Of these, the abundance of Bacteroidetes increased significantly in the AI group, while the abundances of Firmicutes, Proteobacteria, and Actinobacteria decreased significantly in the AI group. Analysis at the class level indicated a decline of Proteobacteria in the AI group, which was mainly attributed to be increased abundance of *Acinetobacter*, while the decline of Firmicutes in the AI group was primarily attributed to the increased abundance of *Weissella* sp. The decline in the abundance of Actinobacteria in the AI group was primarily attributed to *Glutamicibacter* sp. (Figures 3C,D), and the results of LEfSe demonstrated differences in the microbial community structure between the groups (Figure 3E).

Based on the results of community structure analyses, we determined that the microbial flora in the urine samples of the giant pandas in the AI group underwent tremendous changes compared with that of the NM group. The differences were subsequently identified using LDA. The microorganisms with higher relative abundance used as potential markers, and the significance of the different species was analyzed using Mates tats software. The differential strains were screened according to the following criteria: LDA > 4 and *q* < 0.050. At the genus level, we observed that the abundance of *Acinetobacter* sp., *G*. sp., *W*. sp., and *Pseudomonas* sp. increased significantly in the NM group (*p* < 0.05), while the abundance of *L*. sp., *Tissierella* sp., *Romboutsia* sp., and *Sphingopyxis* sp. (Figure 3F).

#### Correlation analysis

**Figure 4:**
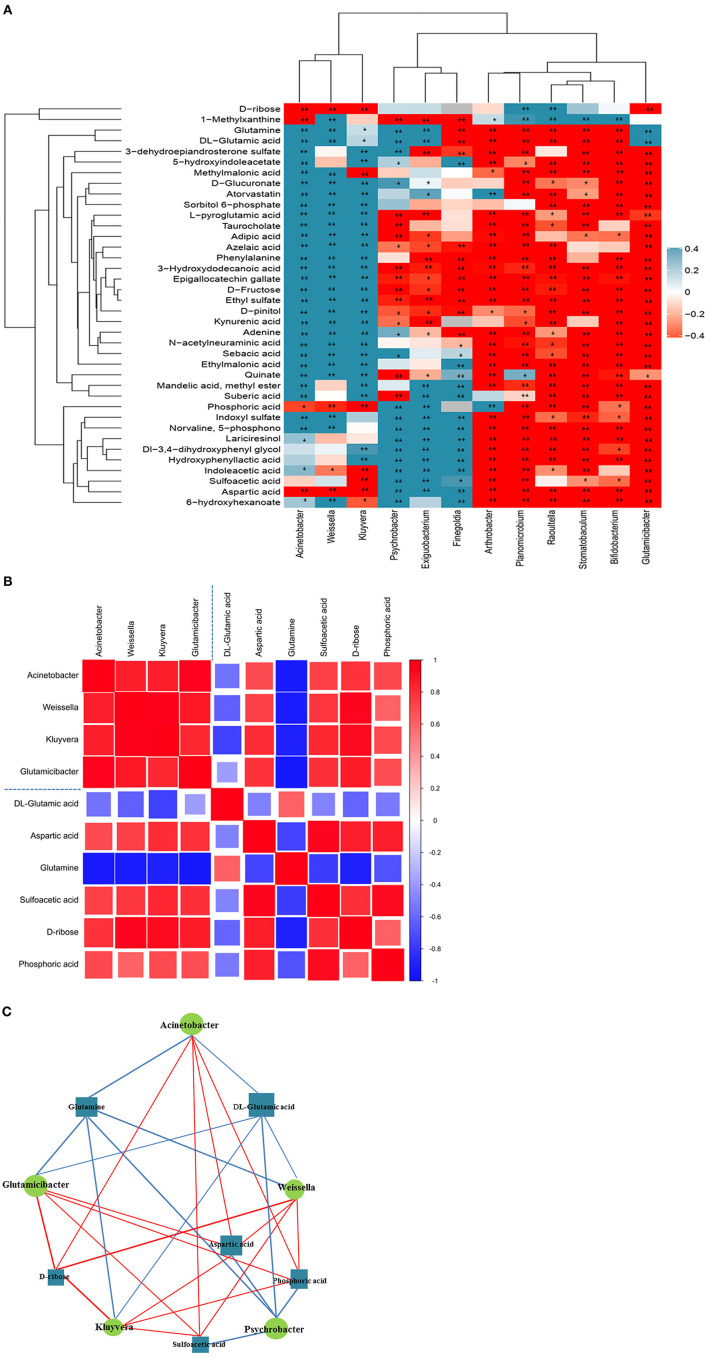
Correlation analysis of urine microorganisms and metabolites. **(A)** Hierarchical clustering heat map of spearman correlation analysis of significant difference between microbiota and metabolites. In the hierarchical clustering heat map, each row represents a significantly different genus, and each column represents a significantly different metabolite. The tree branches on the left represent the results of clustering differential bacterial genera, and the upper tree branches represent the results of clustering analysis on differential metabolites. Clusters of significantly different metabolites or different genera that appear in the same cluster have a similar correlation pattern. Each cell in the hierarchical clustering heat map contains two kinds of information (correlation coefficient *r* and *p*-value). The correlation coefficient r is represented by color. *r* > 0 means positive correlation, which is represented by red; *r* < 0 means negative correlation, which is represented by blue, and the darker the color, the stronger the correlation. *p*-value reflects the significant level of the correlation, *p*-value <0.05, represented by ^*^; *p*-value <0.01, represented by ^**^. **(B)** Matrix heat map of spearman correlation analysis of significant difference between microbiota and metabolites related to arginine, glutamatergic and GABAergic synthesis. The matrix graph not only shows the correlation between the significantly different bacteria and the significantly different metabolites, but also the correlation between the significantly different metabolisms and the significantly different bacteria. Taking the blue dotted line in the picture as the dividing line, the correlation coefficient matrix heat map can be divided into four icons. The upper left corner shows the correlation between significantly different bacterial groups, the lower right corner shows the correlation between significantly different metabolites, and the upper right corner and the lower left corner both show the significantly different bacterial groups and Correlations between significantly different metabolites, mirror symmetry. The Spearman correlation coefficient value r is between −1 and +1. The correlation coefficient r is represented by color. r>0 indicates a positive correlation, which is shown in red. Darker colors indicate stronger correlations. **(C)** Network diagram of spearman correlation analysis of significant difference between microbiota and metabolites related to arginine, glutamatergic and GABAergic synthesis. Circles in the figure represent significantly different genera, and rectangles represent significantly different metabolites. The color of the line represents the positive and negative correlation coefficient values between the two (blue for negative correlation, red for positive correlation), and the thickness of the line is proportional to the absolute value of the correlation coefficient. The size of a node is positively related to its degree, that is, the larger the degree, the larger the node size.

The results of correlation analyses revealed that the altered urinary microbiota was closely related to changes in certain metabolites. The scatter plot revealed that several typical urinary microbiotas were highly related to amino acid metabolites. For instance, *A*. sp., *G*. sp., and *W*. sp. were significantly positively correlated with aspartic acid (*A*. sp.: *r* = 0.790; *p* < 0.01; *G*. sp.: *r* = 0.839; *p* < 0.01; *W*. sp.: *r* = 0.699; *p* < 0.01), sulfoacetic acid (*G*. sp.: *r* = 0.825; *p* < 0.01), and phosphoric acid (*A*. sp.: *r* = 0.559; *p* < 0.05; *G*. sp.: *r* = 0.839; *p* < 0.01; *W*. sp.: *r* = 0.728; *p* < 0.01), but negatively correlated with DL-glutamic acid (*A*. sp.: *r* = −0.797; *p* < 0.01; *G*. sp.: *r* = −0.650; *p* < 0.01; *W*. sp.: *r* = −0.741; *p* < 0.01) and glutamine (*A*. sp.: *r* = −0.682; *p* < 0.01; *G*. sp.: *r* = −0.785; *p* < 0.01; *W*. sp.: *r* = −0.790; *p* < 0.01) (Figure 4A). There was correlation between the significantly altered microbiota and metabolites related to arginine, glutamatergic, and GABAergic synthesis (Figures 4B,C). Notably, the *A*. sp. was the largest in the network diagram of dominant bacteria in the vaginal flora and was positively correlated with the concentration of aspartic acid and negatively correlated with the concentrations of several metabolites, including glutamine and DL-glutamic acid. This suggested that the changes in the concentrations of these metabolites could be attributed to alterations in the abundance of A. sp. (Figures 4B,C).

## Discussion

Previous studies have reported that the low natural reproductive rate of wild animals in captivity, including the giant panda, is widespread (Christie et al., 2012). The factors that affect the natural reproductive efficiency of captive giant pandas are complex and changeable (Zhang et al., 2004; Swaisgood et al., 2006; Li et al., 2017). As giant pandas are wild animal with a strong mate selection behavior, the scientific community believes that the low reproductive success rate of captive giant pandas is related to the loss of their natural mating ability, lack of complete courtship competition and poor sexual desire caused by the human selection of mates as part of the giant panda breeding plan instead of free mating choice (Yan et al., 2021). Compared to other bio fluids, urine is characterized by the richness of metabolites and its ability to reflect imbalances in all biochemical pathways within the body (Owen et al., 2016). We, therefore, employed a UPLC Q-TOF/MS metabolomics technology for identifying metabolic biomarkers in the urine samples of captive giant pandas. The results demonstrated the presence of differentially expressed metabolites between the NM and AI groups. Of these, the metabolites DL-glutamic acid, glutamine, and aspartic acid, which are related to arginine biosynthesis, were significantly altered in giant pandas with poor reproductive ability. This indicated that the decline in the reproductive ability could be related to arginine biosynthesis. This is also consistent with the significantly different metabolic pathways enriched by KEGG pathway analysis based on Fisher's exact test (arginine biosynthesis). The physiologically active form, L-arginine, exerts biological functions in adult animals. Arginine is first converted to citrulline, which is also derived from glutamate in the mitochondria. The produced citrulline combines with aspartic acid and is converted to L-argininosuccinate, which is again converted to arginine (Wu and Morris, 1998). Although there were no significant changes in the urinary content of arginine and argininosuccinate, we observed that the urinary content of aspartic acid, an important intermediate metabolite related to arginine synthesis, was significantly down-regulated in the AI group, while the levels of glutamine and DL-glutamic acid were significantly up-regulated. This indicated that the decline in the reproductive ability of giant pandas is related to the imbalance in arginine synthesis. As glutamate is a key metabolite in several important amino acid anabolic pathways, the physiological demand for glutamine increases under stress. As the synthesis of glutamine does not meet the physiological requirement, glutamate synthesis increases under stress, which competes with and reduces aspartic acid synthesis for meeting the physiological requirement, and in turn reduces arginine synthesis (Scibona et al., 1994; Eskiocak et al., 2006). L-arginine is crucial for the reproductive health of male and female animals. Studies have demonstrated that L-arginine supports normal sperm production and maturation in male animals (Scibona et al., 1994), and simultaneously increases blood flow to the genitals of males and females, leading to increased libido (Moncada and Higgs, 1993; Eskiocak et al., 2006). However, L-arginine deficiency reduces the levels of sex hormones in captive giant pandas due to a decrease in the levels of its metabolic products, including nitrogen oxides and polyamines, which affect the expression of libido and sexual behavior during mate selection (Williams and O'Neill, 2018). By analyzing the data obtained by urinary metabolomics, we also observed that the metabolic pathways in the nervous system (glutamatergic and GABAergic synapses) and amino acid metabolic pathways associated with stress (alanine, aspartate, and glutamate pathway, and D-glutamine, and D-glutamate pathway) in the NM group were also significantly enriched compared to those of the AI group, as revealed by the differential abundance score (Hakimi et al., 2016). The amino acid metabolites, including aspartic acid, GABA, glycine, DL-glutamic acid, and sulfoacetic acid, which are related to inhibitory neurotransmitter pathways, also increased or decreased significantly. Although there is no direct evidence that arginine metabolism is directly related to psychological stress, arginine has a very important positive effect on immune function, which is primarily attributed to increased immunosuppression and the reduction of excessive inflammatory response for maintaining immune balance. In addition, arginine acts as an immune stabilizer (Han et al., 2009). The main metabolites in the arginine metabolic pathway, DL-glutamic acid and glutamine, are also important intermediate metabolites in the synthesis-related pathways of the nervous system (glutamatergic and GABAergic synapses), and alterations in these metabolites are serious signs of psychological stress diseases (Leppik et al., 2018). In this study, we observed that in addition to the arginine biosynthesis pathway, the glutamatergic synapse and GABAergic synapse pathways were significantly annotated, thus confirming the correlation between psychological stress and arginine biosynthesis and metabolism. We therefore speculate that the restriction caused by captivity inhibits the arginine biosynthesis pathway and results in the loss of libido in giant pandas, which subsequently leads to the failure of mate selection. The long-term frustration resulting from mate selection failure induces psychological stress in captive giant pandas, which in turn affects the biosynthesis of arginine.

We subsequently analyzed the differences in the structural diversity of the urinary microbiota of captive giant pandas using a 16S rDNA amplicon sequencing technology. The results demonstrated significant changes in the urinary microbiota at both phylum and genus levels in the AI group. Of these, the relative abundance of Firmicutes in the AI group increased significantly, while the relative abundance of Bacteroidetes decreased significantly. The results of this study were consistent with the results obtained in the study on patients with major depression by Jiang et al. (2015). They observed that the abundance of Firmicutes was relatively lower while the abundance of Bacteroidetes was higher in patients diagnosed with major depressive disorder, compared to those of healthy controls (Jiang et al., 2015). Additionally, studies on intestinal microbiota have demonstrated that severe psychological stress can cause imbalances in the human intestinal flora. The imbalance in intestinal flora caused by alterations in the taxonomic composition of gut microbiota (Firmicutes: Bacteroidetes ratio) leads to the loss of certain metabolites produced by the flora (short-chain fatty acids), which in turn affects the neurotransmitter system of the brain and promotes the onset of psychological stress (Valles-Colomer et al., 2019). The results of 16S rDNA analyses were related to the results obtained by metabolomics analysis, which revealed that the giant pandas in the AI group might have serious psychological stress. We also observed that the relative abundance of Proteobacteria in the human intestinal tract rarely shows variations (Shin et al., 2015); however, the urinary abundance of Proteobacteria in giant pandas accounted for approximately 50% of the total flora. Additionally, the urinary abundance of Proteobacteria in giant pandas in the AI group was significantly lower than that of the NM group. The differences in microbial STAMP between the two groups, determined by Welch's t-test analysis, also revealed that *A*. sp., belonging to the Proteobacteria phylum, exhibited the largest difference in abundance ratio. Although consistent findings have frequently supported this concept, dysbiosis during metabolic disorders often occurs due to an increased prevalence of Proteobacteria. Of the four major phyla (Firmicutes, Bacteroidetes, Proteobacteria, and Actinobacteria), Proteobacteria exhibit the highest intestinal instability over time (Faith et al., 2013). We also observed that the abundance of *P*. sp. increased significantly in the NM group (*p* < 0.05). In a male infertility study, Lundy et al. (2021) observed the abundance of *Prevotella* sp. is inversely associated with sperm concentration, while the abundance of *P*. sp. is directly associated with the total motile sperm count (Lundy et al., 2021). Although this study investigated the relationship between the composition of urinary flora and mating ability, previous studies identified that the diverse semen microbiome has modest similarity to the urinary microbiome. These findings also indicated that the male giant pandas in the AI group have a higher risk of infertility than the male giant pandas in the NM group. Furthermore, the results of 16S rDNA analyses indicated that the decline in natural reproductive capacity had a minor effect on urinary microbiota, with no drastic or subversive changes. It is speculated that dynamic alterations in stress and adaptation occurs in the body during confinement, and the adaptive response mitigates the effect of the reduced natural reproductive capacity on the microecology of urinary microbiota.

The correlation between the altered bacterial genera in the urine and metabolites with altered urinary levels was determined by Spearman correlation analysis. The results demonstrated significant relationships between urinary microorganisms and the levels of urinary metabolites involved in amino acid metabolism and neurotransmitter synthesis. These findings indicated that the alterations in urinary microbiota are related to the alterations in metabolic phenotype. The results of correlation analysis revealed that the *A*. sp., *G*. sp., and *W*. sp. were significantly correlated to metabolites related to arginine biosynthesis, and the glutamatergic and GABAergic synaptic pathways. In particular, we also observed that the content of *P*. sp. in the urine samples of the AI group was significantly lower than that of the NM group. The content of *P*. sp. was also significantly correlated with the metabolites related to arginine synthesis, which further indicated that these bacteria-metabolite pairs are directly or indirectly related to the expression of natural mating behavior. We therefore speculated that the lack of these bacteria would affect the synthesis of hormones, neurotransmitters, and key chemical substances during the mating period, which would in turn affect the sexual desire of the captive giant pandas during the mating period and their mate selection behavior. Although some extrapolations were made in this study, the functional genes could not be identified from the results of 16S amplicon sequencing and urinary metabolite analyses. We intend to employ metagenomics approaches in future studies for interpreting these data.

## Conclusion

Using an UHPLC-TOF/MS metabolomics approach combined with 16S rDNA sequencing techniques, this study demonstrated for the first time that the inhibition of arginine synthesis caused by environmental changes could be related to the poor libido of captive giant pandas during the breeding period. The study also identified the relationship between the urinary abundance of *P*. sp. and levels of metabolites related to arginine synthesis. These findings may aid in understanding the mechanism underlying environment-induced mate selection in captive giant pandas. The results may also aid in the identification of a novel method for determining the sexual desire of giant pandas based on urinary microbiota, which would significantly improve the natural reproductive success rate of captive giant pandas. Targeted metabolomics approaches can be employed in the later stage of research, wherein amino acid, neurotransmitter, and intestinal metagenomics analyses can be performed for identifying and targeting the marker metabolites and flora that affect the mate choice of captive giant pandas. The reconstruction of specific flora or the biosynthetic pathways of key amino acids would aid in improving the natural mating ability of captive giant pandas.

## Data availability statement

The datasets presented in this study can be found in online repositories. The names of the repository/repositories and accession number(s) can be found below: NCBI—PRJNA822516.

## Ethics statement

The study protocol was approved by the Institutional Animal Care and Use Committee of Chengdu Research Base of Giant Panda Breeding (approved number: 2020013).

## Author contributions

M-yZ, RH, Y-lL, and K-lC designed the study and explained the data. M-yZ acquired the funding, performed the experiments, analyzed the data, wrote the manuscript, and reviewed the manuscript. RH and J-hA supervised the study and reviewed the manuscript. X-yW, JA, D-hW, Z-gC, and AJ prepared the samples, helped analyze, review the data, and reviewed the manuscript. All authors contributed to the study conception and design, commented on previous versions of the manuscript, and read and approved the final manuscript.

## Funding

This study was supported by National Nature Science Foundation of China (32100386) and the Program of the Chengdu Research Base of Giant Panda Breeding (2020CPB-B05).

## Conflict of interest

The authors declare that the research was conducted in the absence of any commercial or financial relationships that could be construed as a potential conflict of interest.

## Publisher's note

All claims expressed in this article are solely those of the authors and do not necessarily represent those of their affiliated organizations, or those of the publisher, the editors and the reviewers. Any product that may be evaluated in this article, or claim that may be made by its manufacturer, is not guaranteed or endorsed by the publisher.

## Supplementary material

Supplementary Figure S1 Analysis of significant difference metabolite between NM group and the AI group. **(A)** Volcano plot in positive ion mode. **(B)** Volcano plot in negative ion mode. **(C)** Significant expression analysis of urine metabolites identified by positive ion mode. **(D)** Significant expression analysis of urine metabolites identified by negative ion mode. The abscissa in the figure is the log value of log2 of the Fold Change, and the ordinate is the log value of –log10 of the significance *p*-value. Significantly different metabolites: metabolites that meet FC> 1.5 and *p*-value <0.05 are represented in red, and metabolites that meet FC <0.67 and *p-*value <0.05 are represented in blue. Non-significantly different metabolites are shown in black.

Supplementary Figure S2 Group specific species taxonomic tree. The circles with different colors in the above figure represent the classification level, and the size represents the phase abundance. The circles with different colors in the figure below represent the grouping, and the size represents the proportion of the relative abundance in the classification. The numbers under the circles, the first one indicates the number of sequences that are only aligned to this category (cannot be aligned to the classification level below the category level), and the second number indicates how many sequences are aligned to this category in total.

Supplementary Figure S3 Species abundance cluster heatmap. **(A)** Each sample phylum level. **(B)** Each group phylum level. The horizontal axis represents different samples, and the vertical axis represents different species. The depth of the color is related to the abundance of the species. The darker the color, the higher the abundance is.
