# Supplementary figures and images for: Combined urine metabolomics and 16S rDNA sequencing analyses reveals physiological mechanism underlying decline in natural mating behavior of captive giant pandas
Source: Front Microbiol. 2022 Sep 2;13:906737. doi: 10.3389/fmicb.2022.906737 (PMC9478395; doi:10.3389/fmicb.2022.906737)

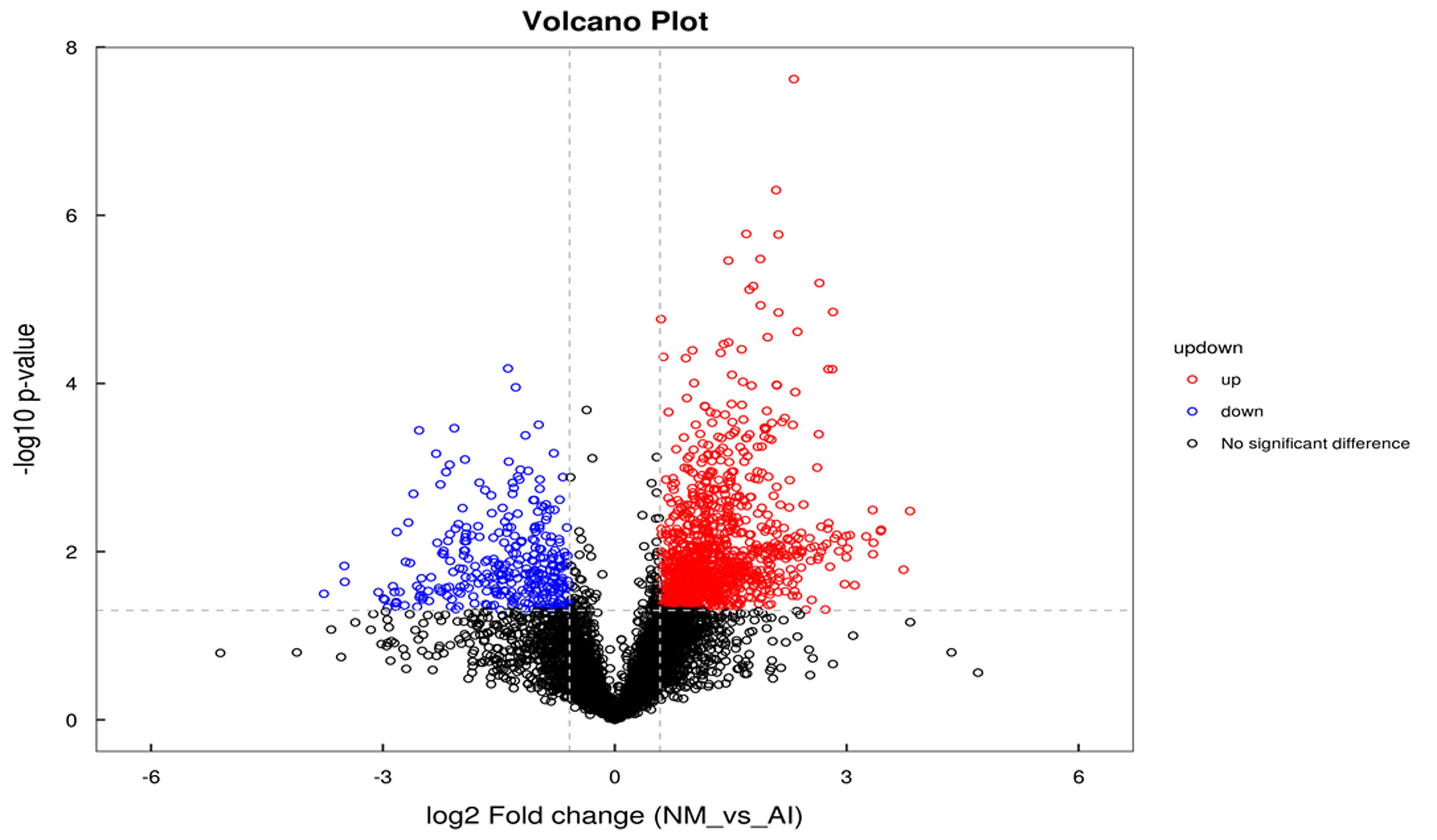

Supplement: Supplementary Figure S1 — Analysis of significant difference metabolite between NM group and the AI group. (A) Volcano plot in positive ion mode. (B) Volcano plot in negative ion mode. (C) Significant expression analysis of urine metabolites identified by positive ion mode. (D) Significant expression analysis of urine metabolites identified by negative ion mode. The abscissa in the figure is the log value of log2 of the Fold Change, and the ordinate is the log value of –log10 of the significance p-value. Significantly different metabolites: metabolites that meet FC> 1.5 and p-value <0.05 are represented in red, and metabolites that meet FC <0.67 and p-value <0.05 are represented in blue. Non-significantly different metabolites are shown in black. [file Image_1.TIF]

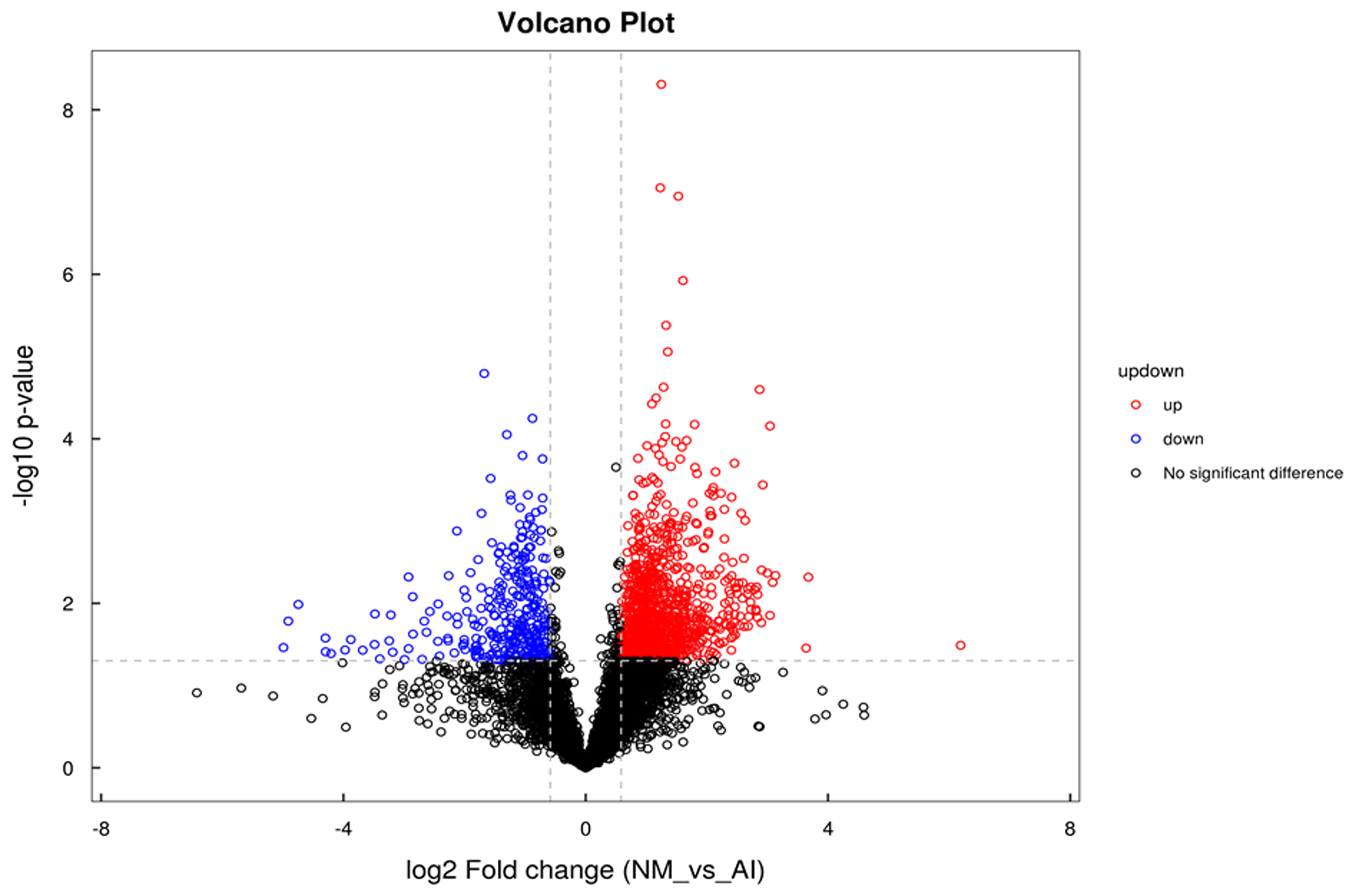

Supplement: Supplementary Figure S2 — Group specific species taxonomic tree. The circles with different colors in the above figure represent the classification level, and the size represents the phase abundance. The circles with different colors in the figure below represent the grouping, and the size represents the proportion of the relative abundance in the classification. The numbers under the circles, the first one indicates the number of sequences that are only aligned to this category (cannot be aligned to the classification level below the category level), and the second number indicates how many sequences are aligned to this category in total. [file Image_2.TIF]

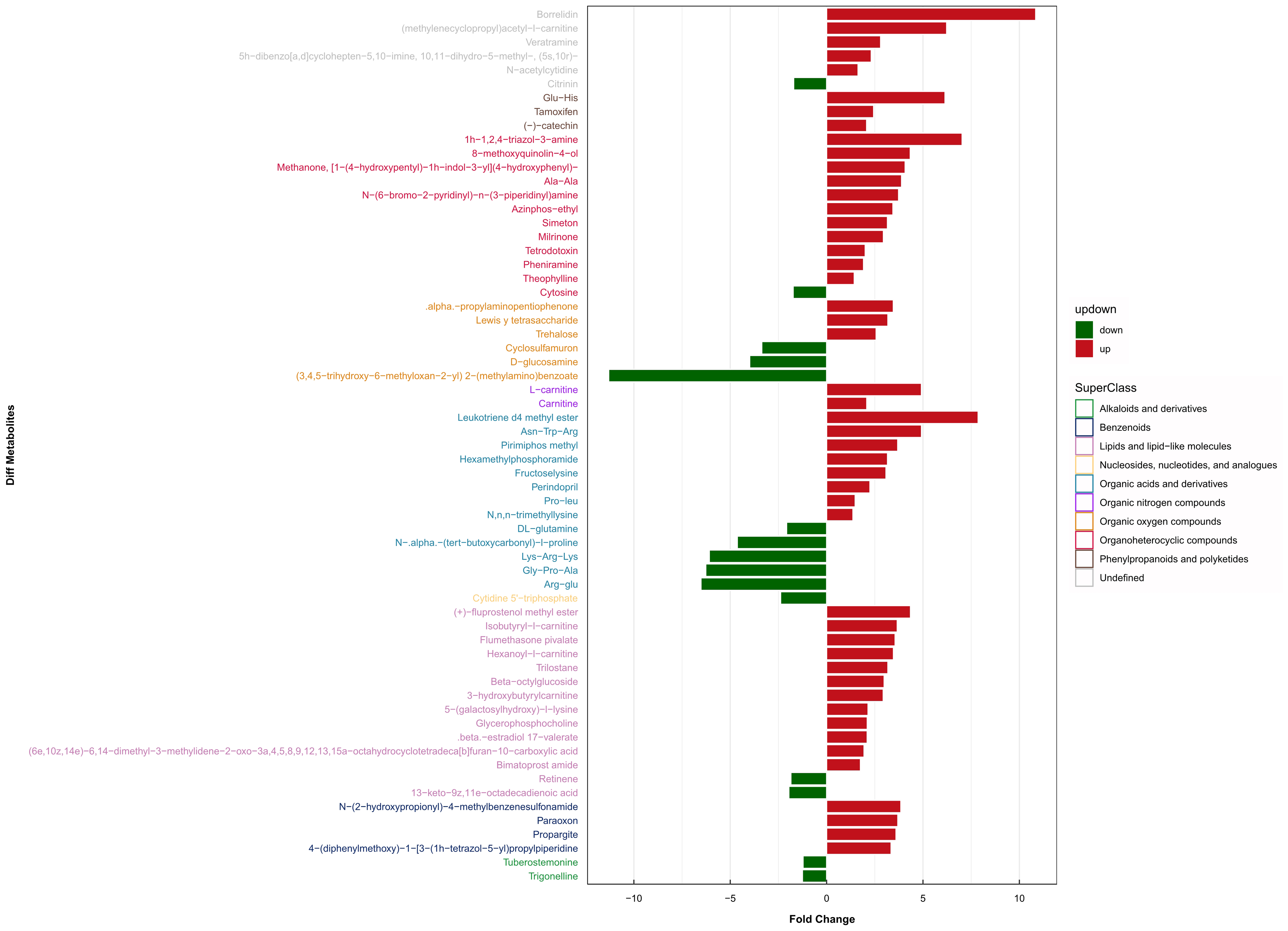

Supplement: Supplementary Figure S3 — Species abundance cluster heatmap. (A) Each sample phylum level. (B) Each group phylum level. The horizontal axis represents different samples, and the vertical axis represents different species. The depth of the color is related to the abundance of the species. The darker the color, the higher the abundance is. [file Image_3.TIF]

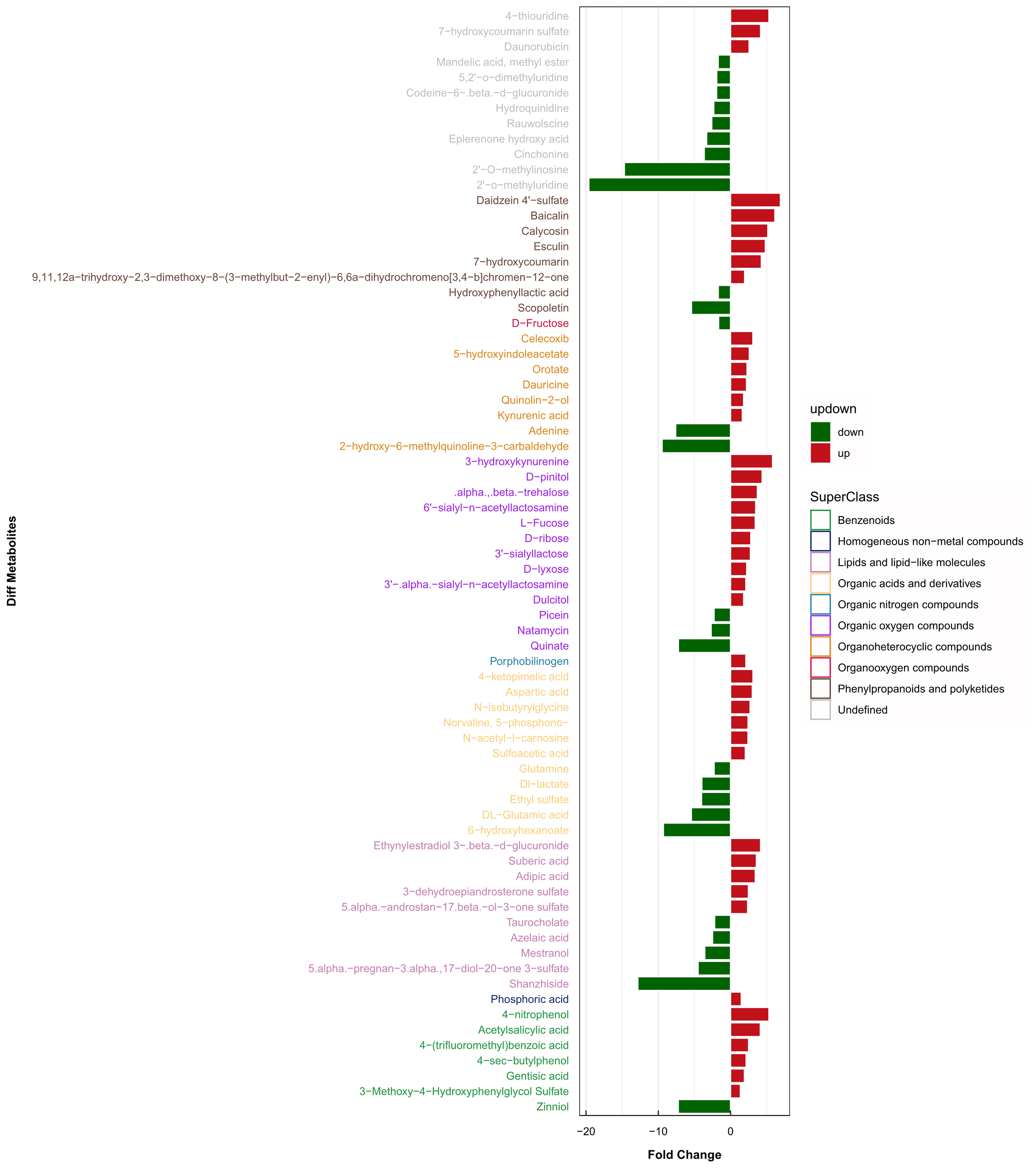

Supplement: Supplementary file 4 [file Image_4.TIF]

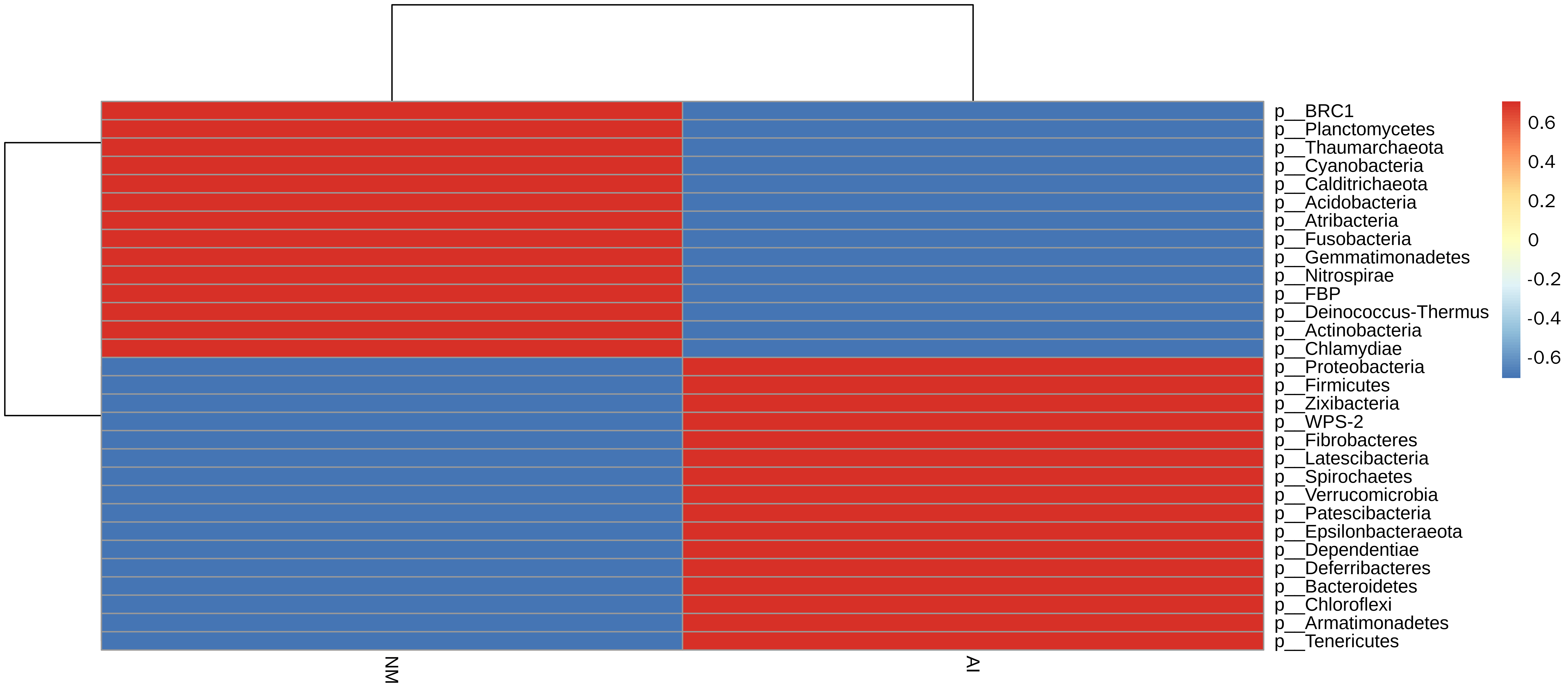

Supplement: Supplementary file 6 [file Image_6.TIF]

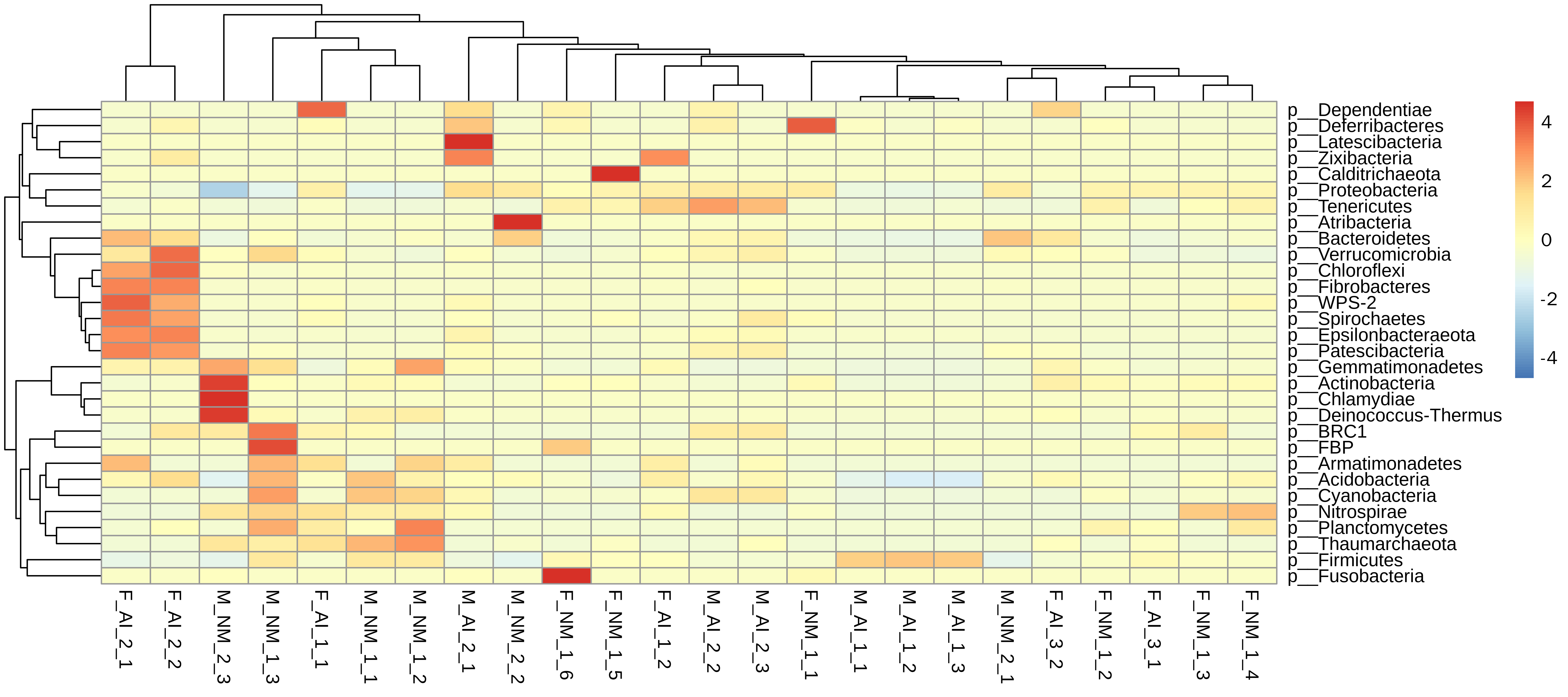

Supplement: Supplementary file 7 [file Image_7.TIF]
